# Supplementary material for: Hardware-in-Loop Comparison of Physiological Closed-Loop Controllers for the Autonomous Management of Hypotension
Source: Bioengineering (Basel). 2022 Aug 27;9(9):420. doi: 10.3390/bioengineering9090420 (PMC9495383; doi:10.3390/bioengineering9090420)
Supplement: Supplementary file 1 [file bioengineering-09-00420-s001.zip › bioengineering-1829468-supplementary.pdf]

Supplementary Information

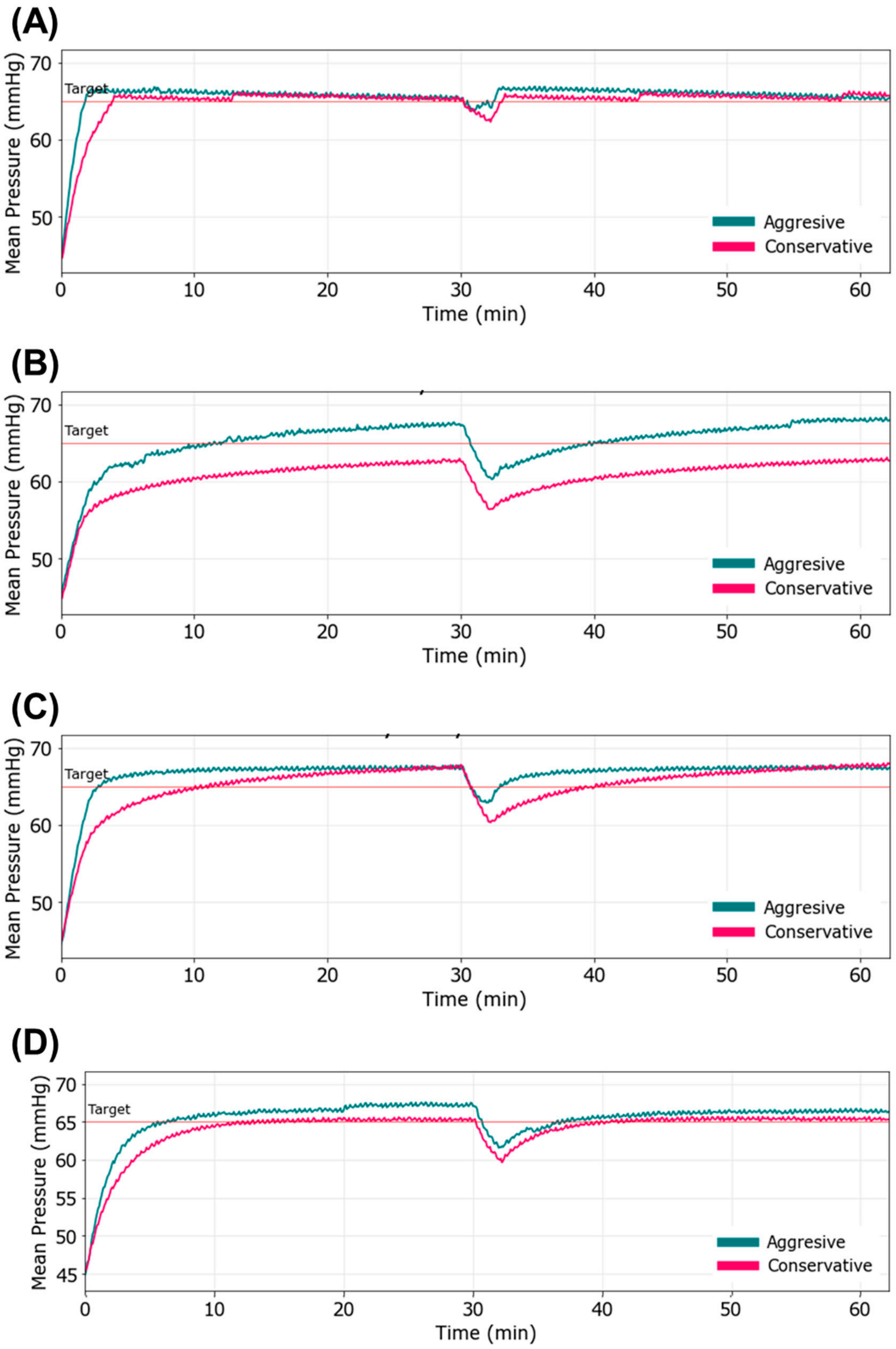

Figure S1. Performance for all Controller Types and Configuration for Scenario 2. Representative MAP vs. time for a single run of the aggressive and conservative (A) Decision Table, (B) Single-input Fuzzy Logic, (C) Dual-input Fuzzy Logic, and (D) PID (proportional-integral-derivative) controllers through Scenario 1. In Scenario 1, MAP began at 45 mmHg with no active hemorrhage until the 30-minute timepoint when a severe hemorrhage occurred for 2 minutes, followed by a return to no hemorrhage.

Table S1. Compilation of metrics for all controller types and configurations for Scenario 1. Types (DT = Decision Table; SFL = Single-input Fuzzy Logic; DFL = Dual-input Fuzzy Logic; PID = proportional-integral-derivative controller) and configurations (Agg = Aggressive; Con = Conservative) vs individual performance metrics. Each individual metric is an average of the normalized values for the three subject variabilities.

	DT:Agg	DT:Con	SFL:Agg	SFL:Con	DFL:Agg	DFL:Con	PID:Agg	PID:Con
MDPE (%)	1.26	0.76	1.63	-5.69	3.33	1.61	1.83	0.16
MDAPE (%)	1.32	0.89	2.74	5.69	3.37	2.73	2.04	0.58
MDAPE_SS (%)	1.30	0.86	2.66	4.96	3.35	2.64	1.99	0.53
Target Overshoot (%)	2.78	2.02	4.85	0.00	4.33	4.62	3.50	1.07
Effectiveness (%)	98.17	96.47	94.73	72.43	97.32	94.68	96.20	92.85
Wobble (%)	0.52	0.32	1.16	0.96	0.31	1.09	0.56	0.35
End-State Divergence (%)	0.79	0.57	0.56	0.67	0.06	0.67	0.05	0.04
Percent Rise Time (%)	1.81	3.20	4.07	7.87	2.55	4.03	3.38	5.51
Volume Efficiency	2.91	3.03	3.18	2.97	3.13	3.18	3.11	3.05
Area Above Target Pressure (%)	1.28	0.78	1.77	0.00	3.04	1.76	1.63	0.29
Area Below Target Pressure (%)	0.46	0.90	1.72	7.07	0.69	1.71	1.11	1.97
Mean Infusion (%)	3.50	3.56	3.77	3.17	3.76	3.77	3.69	3.50
Variable Infusion (%)	71.91	55.58	19.83	24.12	39.66	20.87	87.12	32.10

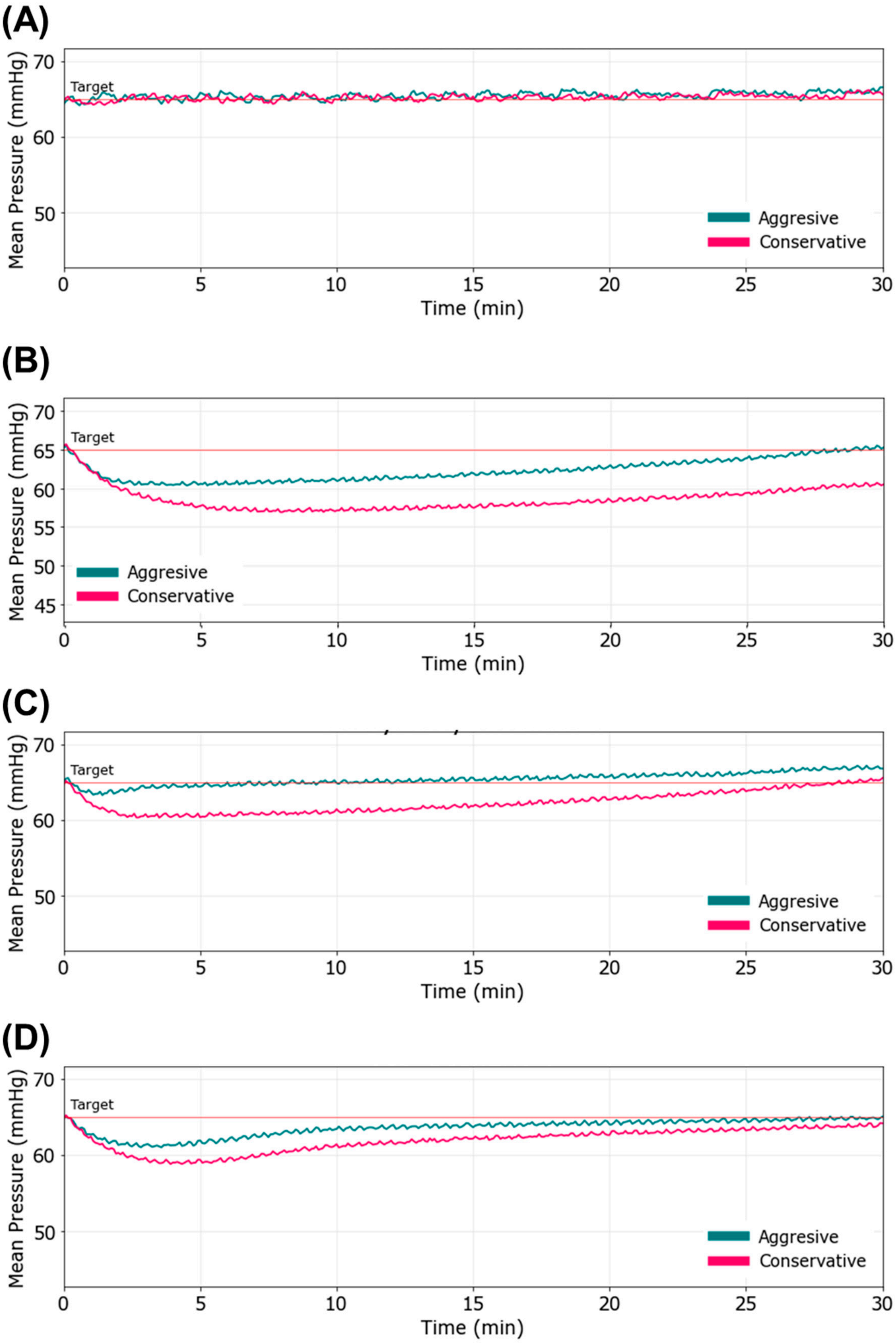

Figure S2. Performance for all Controller Types and Configuration for Scenario 2. Representative MAP vs. time for a single run of the aggressive and conservative (A) Decision Table, (B) Single-input Fuzzy Logic, (C) Dual-input Fuzzy Logic, and (D) PID (proportional-integral-derivative) controllers through Scenario 2. In Scenario 2, MAP begins at a stable 65 mmHg and presents a severe hemorrhage that clots over the 30-minute test scenario.

Table S2. Compilation of metrics for all controller types and configurations for Scenario 2. Types (DT = Decision Table; SFL = Single-input Fuzzy Logic; DFL = Dual-input Fuzzy Logic; PID = proportional-integral-derivative controller) and configurations (Agg = Aggressive; Con = Conservative) vs individual performance metrics. Each individual metric is an average of the normalized values for the three subject variabilities.

	DT:Agg	DT:Con	SFL:Agg	SFL:Con	DFL:Agg	DFL:Con	PID:Agg	PID:Con
MDPE (%)	0.60	0.26	-4.56	-10.39	0.56	-4.52	-1.51	-3.74
MDAPE (%)	0.68	0.45	4.56	10.39	0.93	4.52	1.57	3.74
MDAPE_SS (%)	0.68	0.45	3.66	10.62	0.93	3.61	1.57	3.82
Target Overshoot (%)	2.31	1.72	0.81	0.82	3.41	0.93	1.28	0.53
Effectiveness (%)	100.28	100.28	99.91	16.02	100.28	99.63	100.28	94.72
Wobble (%)	0.48	0.41	1.41	1.10	0.92	1.43	1.13	1.62
End-State Divergence (%)	0.35	0.55	0.78	1.01	0.49	0.83	0.58	0.38
Percent Rise Time (%)	0.00	0.00	0.00	0.37	0.00	0.00	0.00	0.00
Volume Efficiency	1.03	1.01	99.13	76.57	1.04	0.98	100.17	95.53
Area Above Target Pressure (%)	0.68	0.40	0.03	0.00	0.88	0.03	0.13	0.01
Area Below Target Pressure (%)	0.08	0.17	4.14	9.75	0.27	4.11	1.75	3.81
Mean Infusion (%)	6.67	6.48	5.78	4.13	6.64	5.72	7.10	6.52
Variable Infusion (%)	167.19	117.36	8.17	9.78	17.71	8.95	74.72	11.52

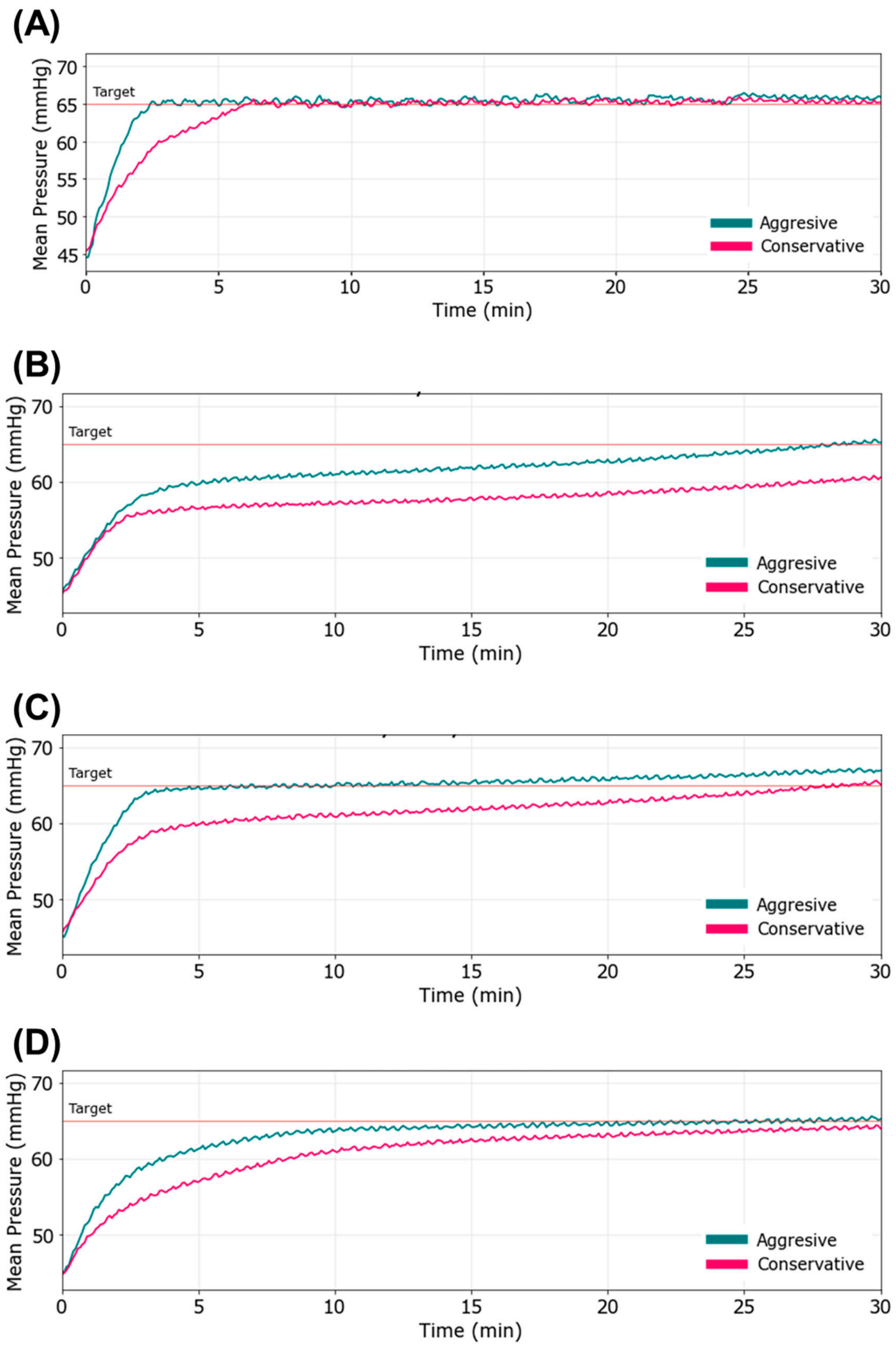

Figure S3. Performance for all Controller Types and Configuration for Scenario 3. Representative MAP vs. time for a single run of the aggressive and conservative (A) Decision Table, (B) Single-input Fuzzy Logic, (C) Dual-input Fuzzy Logic, and (D) PID (proportional-integral-derivative) controllers through Scenario 3. In Scenario 3, MAP began at 45 mmHg with an active hemorrhage that clotted over the 30-minute test run.

Table S3. Compilation of metrics for all controller types and configurations for Scenario 3. Types (DT = Decision Table; SFL = Single-input Fuzzy Logic; DFL = Dual-input Fuzzy Logic; PID = proportional-integral-derivative controller) and configurations (Agg = Aggressive; Con = Conservative) vs individual performance metrics. Each individual metric is an average of the normalized values for the three subject variabilities.

	DT:Agg	DT:Con	SFL:Agg	SFL:Con	DFL:Agg	DFL:Con	PID:Agg	PID:Con
MDPE (%)	0.60	0.23	-4.96	-11.27	0.53	-4.92	-1.01	-3.76
MDAPE (%)	0.74	0.59	4.96	11.27	1.07	4.92	1.38	3.76
MDAPE_SS (%)	0.70	0.46	2.36	9.53	0.89	2.26	1.00	2.11
Target Overshoot (%)	2.27	1.63	0.92	0.00	3.41	0.81	1.97	0.54
Effectiveness (%)	95.09	89.81	81.67	7.22	93.15	81.48	88.89	75.65
Wobble (%)	0.44	0.36	1.16	1.09	0.89	1.23	0.88	1.13
End-State Divergence (%)	0.68	0.25	0.90	1.12	0.49	0.87	0.53	0.35
Percent Rise Time (%)	4.44	8.24	11.57	63.89	6.20	11.11	8.80	17.22
Volume Efficiency	1.84	1.83	1.89	1.73	1.85	1.89	1.79	1.80
Area Above Target Pressure (%)	0.67	0.36	0.02	0.00	0.90	0.03	0.25	0.02
Area Below Target Pressure (%)	1.14	2.23	5.93	11.68	1.58	5.91	3.05	6.00
Mean Infusion (%)	11.01	10.36	9.84	8.16	10.80	9.81	11.40	10.48
Variable Infusion (%)	105.86	69.53	11.97	14.85	19.85	12.33	58.09	13.25

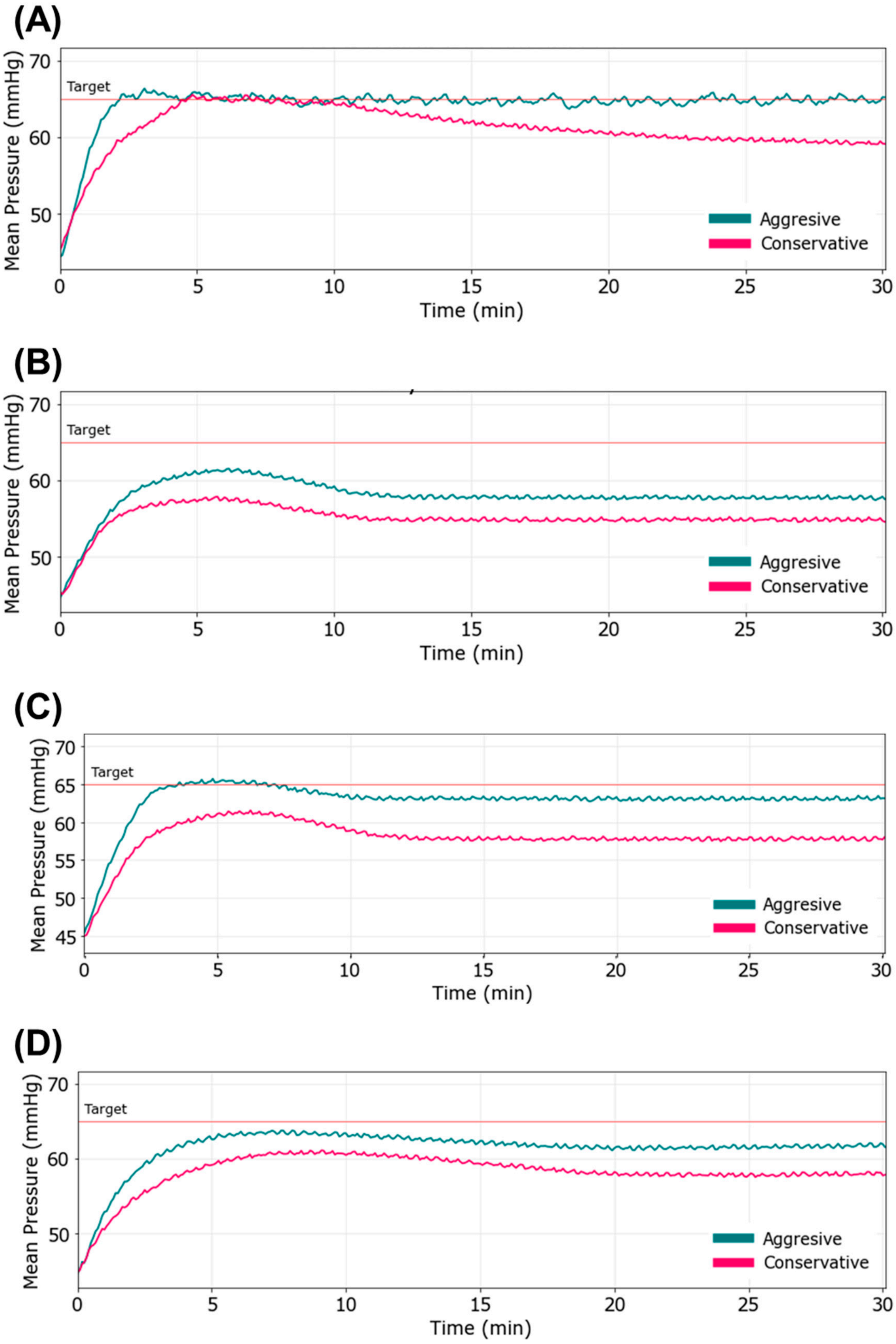

Figure S4. Performance for all Controller Types and Configuration for Scenario 4. Representative MAP vs. time for a single run of the aggressive and conservative (A) Decision Table, (B) Single-input Fuzzy Logic, (C) Dual-input Fuzzy Logic, and (D) PID (proportional-integral-derivative) controllers through Scenario 4. In Scenario 4, MAP began at 45 mmHg with an active hemorrhage that initially clotted until the 5-minute timepoint where clotting mechanisms were halted and hemorrhage rates increased.

Table S4. Compilation of metrics for all controller types and configurations for Scenario 4. Types (DT = Decision Table; SFL = Single-input Fuzzy Logic; DFL = Dual-input Fuzzy Logic; PID = proportional-integral-derivative controller) and configurations (Agg = Aggressive; Con = Conservative) vs individual performance metrics. Each individual metric is an average of the normalized values for the three subject variabilities.

	DT:Agg	DT:Con	SFL:Agg	SFL:Con	DFL:Agg	DFL:Con	PID:Agg	PID:Con
MDPE (%)	-0.29	-6.00	-11.02	-15.55	-2.87	-10.97	-5.16	-10.75
MDAPE (%)	0.56	6.00	11.02	15.55	2.87	10.97	5.16	10.75
MDAPE_SS (%)	0.52	6.70	10.93	15.46	2.80	10.87	5.04	10.51
Target Overshoot (%)	1.66	1.04	0.00	0.00	1.00	0.00	0.00	0.00
Effectiveness (%)	95.66	71.65	15.14	0.00	94.00	14.96	90.95	14.22
Wobble (%)	0.49	1.30	0.37	0.36	0.34	0.38	0.53	0.70
End-State Divergence (%)	0.11	0.47	0.25	0.07	0.11	0.07	0.30	0.17
Percent Rise Time (%)	4.07	7.32	9.17	0.00	5.65	9.91	7.69	12.04
Volume Efficiency	1.23	1.17	1.17	1.15	1.20	1.17	1.20	1.17
Area Above Target Pressure (%)	0.18	0.02	0.00	0.00	0.04	0.00	0.00	0.00
Area Below Target Pressure (%)	1.39	5.99	10.93	15.41	3.48	10.91	5.90	10.95
Mean Infusion (%)	24.34	21.11	18.88	16.58	22.65	18.81	21.19	18.48
Variable Infusion (%)	60.85	12.94	8.69	11.20	12.95	8.79	33.12	7.54

Table S5. Compilation of metrics for all controller types and configurations for averaged across all scenarios. Types (DT = Decision Table; SFL = Single-input Fuzzy Logic; DFL = Dual-input Fuzzy Logic; PID = proportional-integral-derivative controller) and configurations (Agg = Aggressive; Con = Conservative) vs individual performance metrics. Each individual metric is an average of the normalized values for the three subject variabilities across all four scenarios.

	DT:Agg	DT:Con	SFL:Agg	SFL:Con	DFL:Agg	DFL:Con	PID:Agg	PID:Con
MDPE (%)	0.54	-1.19	-4.73	-10.72	0.39	-4.70	-1.46	-4.52
MDAPE (%)	0.83	1.98	5.82	10.72	2.06	5.78	2.54	4.71
MDAPE_SS (%)	0.80	2.12	4.90	10.14	1.99	4.84	2.40	4.24
Target Overshoot (%)	2.26	1.6	1.64	0.2	3.04	1.59	1.69	0.53
Effectiveness (%)	97.30	85.22	72.86	23.92	96.19	72.69	94.08	69.36
Wobble (%)	0.48	0.60	1.02	0.88	0.61	1.03	0.78	0.95
End-State Divergence (%)	0.48	0.46	0.62	0.72	0.29	0.61	0.36	0.23
Percent Rise Time (%)	3.44	6.25	8.27	24.04	4.80	8.35	6.62	11.59
Volume Efficiency	1.75	1.75	1.81	1.65	1.80	1.80	1.78	1.74
Area Above Target Pressure (%)	0.70	0.39	0.45	0.00	1.22	0.45	0.50	0.08
Area Below Target Pressure (%)	0.77	2.32	5.68	10.98	1.51	5.66	2.95	5.68
Mean Infusion (%)	11.38	10.38	9.57	8.01	10.96	9.53	10.85	9.74
Variable Infusion (%)	101.45	63.85	12.16	14.99	22.54	12.74	63.26	16.10
